# Expanding COVID-19 Vaccine Availability: Role for Combined Orthogonal Serology Testing (COST)

**DOI:** 10.3390/vaccines9040376

**Published:** 2021-04-13

**Authors:** Madhusudhanan Narasimhan, Lenin Mahimainathan, Ellen Araj, Andrew E Clark, Kathleen Wilkinson, Sruthi Yekkaluri, Jasmin Tiro, Francesca M Lee, Jyoti Balani, Ravi Sarode, Amit G Singal, Alagarraju Muthukumar

**Affiliations:** 1 Department of Pathology, University of Texas Southwestern Medical Center, Dallas, TX 75390, USA; madhusudhanan.narasimhan@utsouthwestern.edu (M.N.); lenin.mahimainathan@utsouthwestern.edu (L.M.); ellen.araj@utsouthwestern.edu (E.A.); andrew.clark@utsouthwestern.edu (A.EC.); francesca.lee@utsouthwestern.edu (F.ML.); jyoti.balani@utsouthwestern.edu (J.B.); ravi.sarode@utsouthwestern.edu (R.S.); 2 Department of Internal Medicine, University of Texas Southwestern Medical Center, Dallas, TX 75390, USA; kathleen.wilkinson@utsouthwestern.edu (K.W.); sruthi.yekkaluri@utsouthwestern.edu (S.Y.); jasmin.tiro@utsouthwestern.edu (J.T.)

**Keywords:** COVID-19, SARS-CoV-2, IgG, IgM, spike, nucleocapsid, orthogonal antibody testing, vaccine, vaccine prioritization

## Abstract

Background: The persisting Coronavirus disease 2019 (COVID-19) pandemic and limited vaccine supply has led to a shift in global health priorities to expand vaccine coverage. Relying on severe acute respiratory syndrome coronavirus 2 (SARS-CoV-2) molecular testing alone cannot reveal the infection proportion, which could play a critical role in vaccination prioritization. We evaluated the utility of a combination orthogonal serological testing (COST) algorithm alongside RT-PCR to quantify prevalence with the aim of identifying candidate patient clusters to receive single and/or delayed vaccination. Methods: We utilized 108,505 patients with suspected COVID-19 in a retrospective analysis of SARS-CoV-2 RT-PCR vs. IgG-nucleocapsid (IgG_NC_) antibody testing coverage in routine practice for the estimation of prevalence. Prospectively, an independent cohort of 21,388 subjects was simultaneously tested by SARS-CoV-2 RT-PCR and IgG_NC_ to determine the prevalence. We used 614 prospective study subjects to assess the utility of COST (IgG_NC_, IgM-spike (IgM_SP_), and IgG-spike (IgG_SP_)) in establishing the infection proportion to identify a single-dose vaccination cohort. Results: Retrospectively, we observed a 6.3% (6871/108,505) positivity for SARS-CoV-2 RT-PCR, and only 2.3% (2533/108,505) of cases had paired IgG_NC_ serology performed. Prospectively, IgG_NC_ serology identified twice the number of COVID-positive cases in relation to RT-PCR alone. COST further increased the number of detected positive cases: IgG_NC_+ or IgM_SP_+ (18.0%); IgG_NC_+ or IgG_SP_+ (23.5%); IgM_SP_+ or IgG_SP_+ (23.8%); and IgG_NC_+ or IgM_SP_+ or IgG_SP_+ (141/584 = 24.1%). Conclusion: COST may be an effective tool for the evaluation of infection proportion and thus could define a cohort for a single dose and/or delayed vaccination.

## 1. Introduction

The coronavirus disease 2019 (COVID-19) pandemic continues as new variants rise despite a precipitous decline in its threat level since mid-January 2021 in some countries. COVID-19 vaccines have been shown to effectively reduce the risk of symptomatic severe acute respiratory syndrome coronavirus 2 (SARS-CoV-2) infection and associated hospitalizations. However, due to insufficient supply, hesitancy, or burden of the process, a vaccination lag is conspicuous in large segments of “high-priority,” “at-risk,” and other non-priority groups. A majority of the COVID-19 vaccine formulations are approved for use in a two-dose series in the United States and elsewhere [1]. Novel ideas are essential to stretch the vaccine supply and expand vaccination coverage and rates. These advances chiefly depend on the identification of populations that would benefit from a single dose administration. While useful in diagnosing acutely infected individuals, molecular methods cannot identify a significant percentage of untested asymptomatic and mild-to-moderately-symptomatic recovered individuals. Meanwhile, serological testing can identify such individuals during population screening. A recent study found that combined molecular and serological testing improved the diagnosis of both wild-type and D614G spike mutant of SARS-CoV-2 infections in a symptomatic population [2]. Notably, given the low global incidence of reinfection [3,4], some countries including Canada and Italy are already proceeding with a single-dose strategy to expand COVID-19 vaccine access to the public.

Shifting from the mere identification of positive cases to the vaccination setting, an immunological assessment may situationally have a role in informing efficient vaccination strategies. We and others have shown that the immunological response following a primary vaccine dose in SARS-CoV-2 prior-infected (recovered) was comparable to that of two dose-administered naïve population [5,6]. These findings suggest that a natural SARS-CoV-2 infection in recovered individuals acts analogously to the primary vaccine dose among the immunologically naïve, which has led to questions regarding the second dose’s utility. If recovered COVID-19 patients can be considered fully vaccinated following a single dose, the redistribution of the second dose to other populations would improve public health efforts. To address this approach, defining the infection proportion and target populations become pivotal.

Earlier, we demonstrated that the serological assessment of IgG and IgM against SARS-CoV-2 nucleocapsid (_NC_) and spike (_SP_) proteins is reliable in differentiating humoral responses to infection versus vaccination [5,7]. A recent study suggested that a combination of serologic tests can improve the surveillance of low-seroprevalence communities [4]. However, there have been no data linking how a combination orthogonal testing algorithm could help vaccination prioritization. Thus, we sought to address whether a combination orthogonal approach comprising SARS-CoV-2 _NC_ (IgG_NC_) and _SP_-specific IgG (IgG_SP_) and IgM (IgM_SP_) analysis can improve the estimation of previously recovered COVID-19 patients who might benefit from single-dose immunization.

## 2. Materials and Methods

We conducted a retrospective analysis of SARS-CoV-2 RT-PCR and Abbott’s IgG_NC_ antibody testing [7] on 108,505 patients from our healthcare system between 03/2020 and 03/2021 to understand each test’s utilization in routine practice and to estimate the infection proportion. In a separate Dallas/Fort Worth (DFW) metroplex COVID-19 prospective prevalence assessment including 21,388 subjects between 06/2020 and 02/2021, we carried out SARS-CoV-2 RT-PCR and IgG_NC_ estimations on paired blood draws and nasopharyngeal (NP) swabs, as previously described [7]. We used 614 prospective-study non-vaccinated subjects (excluded subjects, *n* = 70) to assess the utility of combined orthogonal serological (IgG_NC_, the recently Food and Drug Administration (FDA)-approved Abbott’s IgM_SP_, and IgG_SP_) testing (COST), as previously described [5]. This study was approved by the University of Texas Southwestern Medical Center’s (UTSW) Institutional Review Board (30630).

## 3. Results and Discussion

Retrospectively, we observed a 6.3% (6871/108,505) positivity for SARS-CoV-2 RT-PCR (Table 1). However, only 2.3% (2533/108,505) of cases had IgG_NC_ performed with a seropositivity of 38.9% (986/2533) (Table 1). Notably, infectious phase-associated fluctuations of viral load influence an assay’s positive detection rate, and combined testing (molecular and serology) modalities have been shown to address this limitation [2,8,9]. This result and the interface of vaccination suggest that an increased volume of serology testing could be worth considering because it could help determine the population-level prevalence of prior infection and accordingly help prioritize vaccination strategies.

In the prospective prevalence analysis, following the exclusion criteria, 21,101 unique subjects were identified. Of these, 646/21,101 (3.1%) were RT-PCR-positive for SARS-CoV-2 (Table 2). However, the IgG_NC_ assay identified more than double the number of positive cases (*n* = 1500/21,101; 7.1%), of which 250 were RT-PCR-positive (Table 2). Excluding RT-PCR-positive cases, 1250 IgG_NC_-positive cases were associated with RT-PCR negativity (1250/20,455 = 6.1%) (Table 2). This showed that all negative PCR results cannot equate to a non-infectious state, and, in contrast, all positive serology results do not necessarily connect with an infectious state; an inconsistency can exist between the PCR- and serology-driven reporting of the size of the actual infected population in a community. Furthermore, these data indicate the utility of serological assessment to maximize the COVID-19 diagnostic yield and to evaluate the prevalence within this population independent of PCR. 

A limitation of using serological analysis as the sole assessment of prevalence is that it can miss individuals in the very early phase of infection or those with waning humoral immunity, leading to underestimation. We and others have demonstrated that the manufacturer-recommended index value cut-off of ≥1.4 for determining positivity using the SARS-CoV-2 IgG assay to nucleocapsid may not be sensitive enough to account precisely for mild and past infections (COVID-19-resolved patients) [5,10,11]. Personal communication between the vendor (Abbott Laboratories, IL, USA) along with our previous study to use the provisional threshold value of SARS-CoV-2 IgG_NC_ (≥0.5–1.39, “grey-zone threshold”; approved in European Union) for clinical use instead of the current FDA-approved ≥1.4 cut-off can help overcome this issue. Chew et al. [10] supported this idea by lowering the IgG_NC_ assay’s positive cut-off and improving the clinical sensitivity of the assay without compromising its specificity. Additionally, we clinically validated an even lower, institution-specific cut-off value of ≥0.2 in correlation with known clinical history and COVID-19 testing using other diagnostic modalities [5]. Among RT-PCR-negative individuals in the DFW prevalence study, applying these two cut-off values increased seropositivity rates to 8.7% (≥0.5) and 12.1% (≥0.2), respectively (Table 2). These findings suggest that lowering the cut-off values can improve the diagnostic yield of early, weak, and waning cases, thereby allowing for the construction and/or refining of a cohort that might qualify for single-dose vaccination. In line with this, a recent study showed that stringent thresholds in SARS-CoV-2 IgG assay can lead to the under-detection of mild infections and the under-estimation of COVID-19 positivity compared to cases identified by clinical symptoms [11]. However, lowering the cut-off’ must be approached with caution in the context of clinical need since it may not maintain sufficient specificity while facilitating the detection of most previous early, weak, and/or mild infections.

The Centers for Disease Control and Prevention (CDC) currently recommends the vaccination of all populations but does not endorse serologic testing to ascertain SARS-CoV-2 infection status [12]. However, when the expected positive-predictive value of a single test is low, which is characteristic of low seroprevalence and waning conditions, the agency does encourage applying one or more tests. This approach called “orthogonal testing” is defined as employing two independent tests in succession, in which the administration of the second test follows the confirmation of the first test’s positivity [12]. To this end, we combined orthogonal class-specific (IgG and IgM), antigenically-unique (SP and NC) serological assays to improve the estimation of early and waning seropositivity, as well as to demonstrate the utility of this approach for identifying patients with previous COVID-19 infections who might benefit from single-dose immunization. Typically, IgG positivity indicates a past infection, while IgM denotes a recent infection.

A total of 614 subjects (excluding confirmed vaccinated or those lacking serological testing by any one assay) underwent the simultaneous measurement of IgM_SP_, IgG_SP_, and IgG_NC_ (Abbott Laboratories), as well as an NP RT-PCR assay [5,7]. The manufacturer-recommended serological positivity thresholds were considered. We observed a greater than three-fold increase in positive cases with individual antibody assays—IgG_NC_ (*n* = 97; 15.8%), IgM_SP_ (*n* = 107; 17.4%), and IgG_SP_ (*n* = 155; 25.2%)—compared to RT-PCR alone (*n* = 30, 4.9%) (Table 3). The reasons for the differential reactivity of IgG_SP_ and IgG_NC_ among unvaccinated patients remain unknown. It is plausible that the IgG_NC_ assay, being qualitative, requires the further refinement of the positive cut-off value. In contrast, the _SP_ assay is quantitative, thus allowing for a dynamic measurement range without modifying the vendor recommended cut-off. Additionally, the _NC_ and _SP_ proteins do not need to bind (avidity) equally the germline-encoded precursors, thus leading to varied positivity rates [4].

Among SARS-CoV-2 RT-PCR-negative subjects, COST data showed a further increase in the number of positive cases: either IgG_NC_+ or IgM_SP_+ (18.0%); either IgG_NC_+ or IgG_SP_+ (23.5%); either IgM_SP_+ or IgG_SP_+ (23.8%); and either IgG_NC_+ or IgM_SP_+ or IgG_SP_+ (141/584 = 24.1%) (Table 3). This suggested that COST, rather than a single test alone, can further improve the identification of previously infected individuals (which may include early and waning subjects), thus forming a relatively reliable foundation to establish a single-dose vaccination cohort. This, in turn, can inform vaccine allocation programs and front-line public health practitioners when setting priorities. Recently, Bubar et al. [13] modeled and reported that the incorporation of serological testing and population seroprevalence could efficiently help prioritize vaccination across countries [13].

Further applying this orthogonal serology algorithm across our healthcare system, a higher proportion of seropositivity (COVID-19 infection estimates) is likely. These findings have notable implications when considering the long-term consequences of the pandemic, particularly regarding the post-acute sequelae of COVID-19. These data are also important for a veritable quantification of disease prevalence that can help segregate a cohort for vaccine prioritization. While serologic assays situationally facilitate the determination of the true magnitude of an outbreak by mapping the epicenter and vulnerable populations [14], their utility in aiding the design and deployment of SARS-CoV-2 immunization and allocation strategies is only beginning to be appreciated. At the same time, such serology testing could also complicate and delay vaccination coverage because it may require additional visits to diagnostic facilities.

## 4. Conclusions

In sum, COST both provides information concerning historical COVID-19 infection status and helps to identify cohorts that can cope with a skipped and/or delayed second dose of the COVID-19 vaccine. In this context, this approach can facilitate the inoculation of a broader population and accelerate vaccine rollout to groups that might otherwise lack access to these therapeutics.

## Figures and Tables

**Table 1:** Retrospective analysis of the severe acute respiratory syndrome coronavirus 2 (SARS-CoV-2) IgG nucleocapsid assay (IgG_NC_) performed in relation to the RT-PCR assay in our health system. COVID-19: coronavirus 2019.

Information	*n* (%)
Total UTSW PCR orders	108,505
COVID-19 PCR+	6871/108,505 (6.3)
COVID-19 PCR–	101,634/108,505 (93.6)
Total UTSW IgG_NC_ orders	2533
IgG_NC_+	986/2533 (38.9)
IgG_NC_–	1547/2533 (61.1)
IgG_NC_ orders against total PCR orders	2533/108,505 (2.3)

**Table 2:** Prospective comparison of SARS-CoV-2 RT-PCR with the IgG nucleocapsid assay (at different cut-off levels) for determining the ‘infection proportion’ in a unique Dallas/Fort Worth metroplex general population.

PCR Status	Information/Explanations	*n* (%)
	Total patients tested	21,388
	Excluded: Confirmed vaccinated, no paired PCR or IgG_NC_ results)	287/21,388 (1.3)
PCR+		646/21,101 (3.1)
PCR-		20,455/21,101 (96.9)
PCR+ and PCR–	Manufacturer-recommended IgG_NC_+ (≥1.4)	1500/21,101 (7.1)
PCR+	Manufacturer-recommended IgG_NC_+ (≥1.4)	250/21,101 (1.2)
PCR–	Manufacturer-recommended IgG_NC_+ (≥1.4)	1250/20,455 (6.1)
	Manufacturer-recommended grey-zone IgG_NC_+ threshold approved in Europe (≥0.5) ^1^	1789/20,455 (8.7)
	UTSW IgG_NC_+ threshold that accounts for exCOVID-19 cases (≥0.2 to <1.4) ^2^	2475/20,455 (12.1)

^1^ Personal communication with the vendor (can be shared upon request). ^2^ Narasimhan et al., (2021) [5].

**Table 3:** Combination orthogonal serological testing (COST) with alternative cut-off for IgG_NC_ assay in comparison with SARS-CoV-2 RT-PCR testing to determine the ‘infection proportion’ in prospective recent Dallas/Fort Worth (DFW) samples. SP: spike.

PCR Status	Information	*n* (%)
	Total patients tested	684
	Excluded: confirmed vaccinated and no information for any one of the antibody assays	70 (10.2)
PCR+		30/614 (4.9)
PCR-		584/614 (95.1)
PCR+ and PCR–	IgG_NC_+ (≥1.4)	97/614 (15.8)
	IgM_SP_+ (≥1.0)	107/614 (17.4)
	IgG_SP_+ (≥50.0)	155/614 (25.2)
PCR–	IgG_NC_+ (≥1.4)	78/584 (13.4)
	Grey-zone IgG_NC_+ (≥0.5)	100/584 (17.1)
	≥UTSW IgG_NC_+ (≥0.2)	130/584 (22.3)
	Either IgG_NC_+ or IgM_SP_+	105/584 (18.0)
	Either IgG_NC_+ or IgG_SP_+	137/584 (23.5)
	Either IgM_SP_+ or IgG_SP_+	139/584 (23.8)
	Either IgG_NC_+ or IgM_SP_+ or IgG_SP_+	141/584 (24.1)

## Data Availability

Due to ethical reasons, the data are not publicly available. However, upon request, the data presented in this study can be shared.

## References

[1] Burgos R.M., Badowski M.E., Drwiega E., Ghassemi S., Griffith N., Herald F., Johnson M., Smith R.O., Michienzi S.M. (2021). The race to a COVID-19 vaccine: Opportunities and challenges in development and distribution. Drugs Context.

[2] Mlcochova P., Collier D., Ritchie A., Assennato S.M., Hosmillo M., Goel N., Meng B., Chatterjee K., Mendoza V., Temperton N. (2020). Combined point-of-care nucleic acid and antibody testing for SARS-CoV-2 following emergence of d614g spike variant. Cell Rep. Med..

[3] Fiore B.D., Paola L., Eugenio M., Gaetano B., Anna V., Antonella L., Annalisa S., Laura M. (2021). Anti-spike S1 receptor-binding domain antibodies against SARS-CoV-2 persist several months after infection regardless of disease severity. J. Med. Virol..

[4] Ripperger T.J., Uhrlaub J.L., Watanabe M., Wong R., Castaneda Y., Pizzato H.A., Thompson M.R., Bradshaw C., Weinkauf C.C., Bime C. (2020). Orthogonal SARS-CoV-2 serological assays enable surveillance of low-prevalence communities and reveal durable humoral immunity. Immunity.

[5] Narasimhan M., Mahimainathan L., Raj E., Clark A.E., Markantonis J., Green A., Xu J., SoRelle J.A., Alexis C., Fankhauser K. (2021). Clinical evaluation of the Abbott Alinity SARS-CoV-2 spike-specific quantitative IgG and IgM assays in infected, recovered, and vaccinated groups. J. Clin. Microbiol..

[6] Mazzoni A., Di Lauria N., Maggi L., Salvati L., Vanni A., Capone M., Lamacchia G., Mantengoli E., Spinicci M., Zammarchi L. (2021). First dose mRNA vaccination is sufficient to reactivate immunological memory to SARS-CoV-2 in ex COVID-19 subjects. MedRxiv.

[7] Phipps W.S., SoRelle J.A., Li Q.Z., Mahimainathan L., Araj E., Markantonis J., Lacelle C., Balani J., Parikh H., Solow E.B. (2020). SARS-CoV-2 antibody responses do not predict COVID-19 disease severity. Am. J. Clin. Pathol..

[8] Wang W.L., Xu Y.L., Gao R.Q., Lu R.J., Han K., Wu G.Z., Tan W.J. (2020). Detection of SARS-CoV-2 in different types of clinical specimens. JAMA.

[9] Cai X.F., Chen J., Hu J.L., Long Q.X., Deng H.J., Liu P., Fan K., Liao P., Liu B.Z., Wu G.C. (2020). A peptide-based magnetic chemiluminescence enzyme immunoassay for serological diagnosis of coronavirus disease 2019 (COVID-19). J. Infect. Dis..

[10] Chew K., Tan S., Saw S., Pajarillaga A., Zaine S., Khoo C., Wang W., Tambyah P., Jureen R., Sethi S. (2020). Clinical evaluation of serological IgG antibody response on the Abbott Architect for established SARS-CoV-2 infection. Clin. Microbiol. Infect..

[11] Eyre D.W., Lumley S.F., O’Donnell D., Stoesser N.E., Matthews P.C., Howarth A., Hatch S.B., Marsden B.D., Cox S., James T. (2021). Stringent thresholds in SARS-CoV-2 IgG assays lead to under-detection of mild infections. BMC Infect. Dis..

[12] CDC (2020). Interim Guidelines for Covid-19 Antibody Testing. https://www.cdc.gov/coronavirus/2019-ncov/lab/resources/antibody-tests-guidelines.html.

[13] Bubar K.M., Reinholt K., Kissler S.M., Lipsitch M., Cobey S., Grad Y.H., Larremore D.B. (2021). Model-informed COVID-19 vaccine prioritization strategies by age and serostatus. Science.

[14] Peeling R.W., Wedderburn C.J., Garcia P.J., Boeras D., Fongwen N., Nkengasong J., Sall A., Tanuri A., Heymann D.L. (2020). Serology testing in the COVID-19 pandemic response. Lancet Infect. Dis..

